# Modulation of Heart Rate Variability and Brain Excitability through Acute Whole-Body Vibration: The Role of Frequency

**DOI:** 10.5114/jhk/183745

**Published:** 2024-04-25

**Authors:** Jingwang Tan, Jianbin Lei, Sam S. X. Wu, Roger Adams, Xueping Wu, Qingwen Zhang, Lijiang Luan, Jia Han, Yu Zou

**Affiliations:** 1 Department of Sport and Exercise Science, College of Education, Zhejiang University, Hangzhou, China.; 2 School of Health Sciences, University of Tasmania, Launceston, TAS, Australia.; 3 College of Rehabilitation Sciences, Shanghai University of Medicine and Health Sciences, Shanghai, China.; 4 Research Institute for Sport and Exercise, University of Canberra, Canberra, Australia.; 5 School of Physical Education, Shanghai University of Sport, Shanghai, China.; 6 School of Exercise and Health, Shanghai University of Sport, Shanghai, China.; 7 Faculty of Health, Arts and Design, Swinburne University of Technology, Hawthorn, VIC, Australia.

**Keywords:** whole-body vibration, frequency, heart rate variability, brain excitability, neuronal entrainment

## Abstract

This cross-over study aimed to explore effects of acute whole-body vibration (WBV) at frequencies of 5–35 Hz on heart rate variability and brain excitability. Thirteen healthy physically active college students randomly completed eight interventions under the following conditions: static upright standing without vibration (CON), static squat exercise (knee flexion 150°) on the vibration platform (SSE), and static squat exercise (knee flexion 150°) combined with WBV at vibration frequency of 5, 9, 13, 20, 25, and 35 Hz. Five bouts × 30 s with a 30-s rest interval were performed for all interventions. The brain’s direct current potentials (DCPs), frequency domain variables (FDV) including normalized low frequency power (nLF), normalized high frequency power (nHF) and the ratio of LF to HF (LF/HF), along with the mean heart rate (MHR) were collected and calculated before and after the WBV intervention. Results suggested that WBV frequency at 5 Hz had a substantial effect on decreasing DCPs [−2.13 μV, t(84) = −3.82, p < 0.05, g = −1.03, large] and nLF [−13%, t(84) = −2.31, p = 0.04, g = −0.62, medium]. By contrast, 20–35 Hz of acute WBV intervention considerably improved DCPs [7.58 μV, t(84) = 4.31, p < 0.05, g = 1.16, large], nLF [17%, t(84) = 2.92, p < 0.05, g = 0.79, large] and the LF/HF [0.51, t(84) = 2.86, p < 0.05, g = 0.77, large]. A strong (r = 0.7, p < 0.01) correlation between DCPs and nLF was found at 5 Hz. In summary, acute WBV at 20–35 Hz principally activated the sympathetic nervous system and increased brain excitability, while 5-Hz WBV activated the parasympathetic nervous system and reduced brain excitability. The frequency spectrum of WBV might be manipulated according to the intervention target on heart rate variability and brain excitability.

## Introduction

As a form of neuromuscular training, whole-body vibration (WBV) has been widely researched in the area of sports science and medicine. Underlying the WBV benefits, the effects of WBV on heart rate variability (HRV) and brain excitability (BE) play a modulating role. For instance, HRV changes induced by WBV have been found to be associated with alterations of blood pressure among obese postmenopausal women (Wong et al., 2016), while changes of BE in WBV intervention could affect central motor commands and subsequently cause kinematic or functional changes (Cochrane, 2011). Frequency, one of the important variables in the WBV program, potentially influences the effects of WBV on HRV and BE. Based on previous evidence, WBV at 15 Hz was shown to be superior to 5 Hz in altering HRV when conducted in a sitting posture (Liu et al., 2021), whereas acute WBV at 27 Hz rather than 10 Hz tended to induce higher cortical activation (Choi et al., 2019). In this sense, the effects of WBV frequencies on HRV and BE may play a mediator role in WBV benefits. Adjustments of frequency may influence practical application of WBV such as accelerating recovery after exercise for athletes (Liu et al., 2021) and improving cardiorespiratory responses among patients with chronic obstructive pulmonary disease (Pleguezuelos et al., 2018).

To date, there has been little evidence regarding the effects of WBV at different frequencies on HRV, BE, and the connection between HRV and BE. Previous studies investigating these questions either used a narrow spectrum of frequency (Choi et al., 2019) or performed WBV in the seated posture (Jiao et al., 2004; Liu et al., 2021), which may provide limited understanding of the function of WBV exercise that is normally conducted in a standing posture. Additionally, despite the reported evidence of WBV on drowsiness in the driving circumstance (Bhuiyan et al., 2022; Zhang et al., 2018), it seemed that effects of low frequency WBV (below 10 Hz) on HRV and BE have been neglected to some extent in the field of sport science and medicine. On the other hand, although previous studies explored the neurophysiological reaction (e.g., corticospinal excitability) to WBV (Krause et al., 2016; Mileva et al., 2009), the mechanism of WBV frequency has not been clearly elucidated.

In this study, we aimed to explore the effects of acute WBV at different frequencies (i.e., 5–35 Hz) on HRV and BE. Furthermore, we also investigated the correlation of changes between HRV and BE with the change of frequencies. The evaluation of effects of WBV frequency on HRV and BE may not only provide insight into understanding the neurophysiological mechanism of WBV on humans, but also extend the applicability of WBV at different frequencies. We hypothesized that acute WBV intervention at different frequencies would have various effects on HRV and BE, and that there would be significant correlations between changes of HRV and BE at different frequencies of acute WBV.

## Methods

### Participants

As a part of a research protocol registered at the Chinese Clinical Trial Registry (ChiCTR2300068972) on 02 March 2023, this study was performed in accordance with the Declaration of Helsinki. The needed sample size was calculated using the G*power software package (version 3.1.9, Heinrich-Heine-Universität Düsseldorf, Düsseldorf, Germany). The total sample size of 13 was estimated from the software. Inclusion criteria used in the recruitment process were as follows: 1) voluntary participation in the experiment; 2) no history of WBV exercise experience. The following exclusion criteria were applied: 1) contraindication to WBV intervention (e.g., acute musculoskeletal injury, pregnancy, pacemaker, etc.); 2) diseases or medication intake that might affect HRV (e.g., cardiovascular diseases, diabetes, adrenoreceptor agonists, etc.) and BE (e.g., neurological diseases, psychological disorders, antidepressant drug treatment, etc.). Eventually, a total of 13 healthy physically active college students (8 men, 5 women; age: 25.1 ± 3.7 years for males, 19.8 ± 0.8 years for females; body height: 177.1 ± 6.1 cm for males, 166.8 ± 3.7 cm for females; body mass: 73.9 ± 12.1 kg for males, 57.1 ± 7.3 kg for females; BMI: 23.5 ± 2.9 kg/m^2^ for males, 20.6 ± 2.1 kg/m^2^ for females) at the Zhejiang University were recruited. Each participant signed informed consent before participating in the experiment.

### Measures

In this study, BE was evaluated by measuring the brain’s direct current potentials (DCPs) which denote sustained shifts and slow deflections (0–0.5 Hz) of cerebral potentials, but are usually superimposed with conventional electroencephalography waves (0.5 Hz or higher) (Valenzuela et al., 2022). DCPs are correlated with brain functions and processes and they can be used to reflect alterations of the excitation in the cerebral cortex and subcortical structures (Kovac et al., 2018). The data of HRV and BE were collected through the Omegawave Diagnostic System (Omegawave Ltd, Espoo, Finland), which had been tested to be reliable and valid (Naranjo-Orellana et al., 2021; Valenzuela et al., 2022). The procedure of assessment was structured in accordance with a previously published checklist (Catai et al., 2020). All tests were evaluated before and after the WBV intervention in a dark, quiet, separate room with suitable temperature (around 26 degrees Celsius) and humidity (approximate 45%). An independent researcher who was blinded to the allocation of participants conducted the assessment. Familiarization was performed during the first visit. Prior to testing, participants were asked to avoid caffeine, alcohol, stress, excessive food intake and moderate to vigorous physical activities.

### Design and Procedures

**Figure 1:**
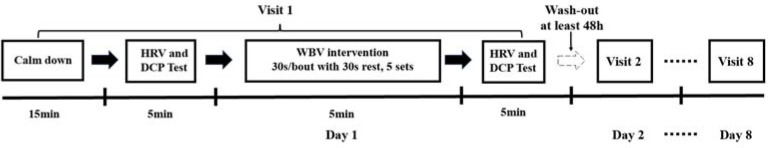
Flowchart demonstrating the whole-body vibration intervention procedure. DCP, direct current potential; HRV, heart rate variability; WBV, whole-body vibration

The current study was conducted following a single-blind randomized controlled cross-over design protocol. There were a total of eight conditions including static upright standing without vibration (CON), static squat exercise (knee flexion 150°) on the vibration platform (SSE), and static squat exercise (knee flexion 150°) combined with WBV at vibration frequency of 5, 9, 13, 20, 25, and 35 Hz. The whole procedure of the experiment is presented in Figure 1. To accomplish the experiment, participants were asked to visit the fitness center eight times with a wash-out period lasting for at least 48 h between each test. Before the initial test, demographic information was entered into the system software. When arrived, participants were told to sit quietly in a chair to calm down for at least 15 min before the assessor conducted the evaluation process. The chest strap of the system was initially soaked in water and then was placed around the torso. Then, the sensor of the system was manually attached with the strap. Two electrodes, connected with sensors with a micro-USB port, were placed at the middle of the forehead and at the base of the thumb. Once prepared, participants were asked to lie on the treatment bed in a supine position. The recording period for the baseline value was 5 minute with a sampling rate of 500 Hz. During the test, participants were asked not to talk, sleep or move the body during the data collection. In addition, they were instructed to minimize distraction and emotional fluctuations. Following the test procedure, participants were asked to report events such as distraction, sneeze, cough, and postural changes, which were noted down during data collection.

Immediately following the first assessment, participants were guided to receive the WBV intervention program, which was conducted by a professional trainer in the fitness center according to previously published guidelines (van Heuvelen et al., 2021). Two synchronous (A6, TiFit, China; Pro 5, Power Plate, USA) and one side-alternating apparatus (S25, Galileo, Germany) producing constant sinusoidal vibrations were used in this study. The vibration parameters were calibrated by an accelerometer (66B, VICTOR Corp., China; sampling rate 1 Hz) that was firmly placed at the central point of the platform for synchronous vibration and at the middle point between the second and the third toe of the forefeet for the side-alternating device. Prior to the intervention procedure, participants who wore flat shoes were given verbal instructions regarding the procedure of the intervention. Then, they were instructed to stand on the vibration platform placed on the ground with their eyes looking forward, hands placed on a handrail, and body mass evenly distributed on both flat feet. The distance between feet was aligned with shoulder width in synchronous devices, while this value was adjustable based on the targeted vibration magnitude in the side-alternating equipment. A static squat position with knee flexion at 150° (considering 180° as full knee extension) calibrated by a goniometer was adopted to reduce transmission of vibration to the head. No additional load or supplementary tool were added during the intervention program. Participants performed the WBV intervention at different frequencies (5, 9, 13 Hz in TiFit A6; 20, 25 Hz in Galileo S25; and 35 Hz in Power Plate Pro 5) with the corresponding peak acceleration (0.1, 0.3, 0.7 g in TiFit A6; 2.5, 3.9 g in Galileo S25; and 4.9 g in Power Plate Pro 5) (Table 1) in random order. In order to avoid potential fatigue, the WBV session was made up of five 30-s bouts with 30-s rest intervals in between. Participants in the static squat exercise (SSE) group performed the same exercise on the vibration platform, but with the machine turned off, whereas participants in the control group (CON) just stood upright on the vibration device without any form of exercise.

**Table 1:** Frequency, peak-to-peak displacement and peak acceleration of the whole-body vibration equipment.

	Manufacturer (vibration type)					
	TIFIT (Synchronous)			Galileo (Side-alternating)		Power Plate (Synchronous)
Frequency (Hz)	5	9	13	20	25	35
Peak-to-peak displacement (mm)	2	2	2	3.15	3.15	2
Peak acceleration (g)	0.1	0.3	0.7	2.5	3.9	4.9

To provide a more reliable comparison of frequency components across studies and reduce the variability of methodological differences, frequency domain variables (FDV) including normalized low frequency power (nLF) and normalized high frequency power (nHF) were selected (Pham et al., 2021). Additionally, balance of the autonomic nervous system (ANS) was estimated by the ratio of low frequency power to high frequency power (LF/HF), in which LF/HF > 1, < 1 and 1 indicated sympathetic nervous system (SNS) dominance, parasympathetic nervous system (PNS) dominance and sympathetic-parasympathetic balance, respectively (Laborde et al., 2017). Changes of the mean heart rate (MHR) were used to assess cardiovascular responses caused by the WBV intervention. DCPs were automatically calculated through the Omegawave system. The above results were manually extracted from the software for subsequent analyses.

### Statistical Analysis

The SPSS software package (Version 26.0, SPSS Inc., Chicago, IL, USA) and Jamovi 2.3.26 were used for statistical analyses. The normality of the data was evaluated using the Shapiro-Wilk test. One factor repeated measures analysis of variance was used to examine the main effect of frequency. Partial eta squared (η2 p) ≥ 0.26, 0.13–0.25, and 0.02–0.12 denoted large, medium, and small effect size, respectively. A post-hoc analysis was performed using paired Student's *t* tests with Tukey adjustment if a significant main effect was found. The effect size was calculated with the Hedges’g value in which < 0.1, 0.1–0.34, 0.35–0.64, 0.65–1.19, and ≥ 1.20 were considered trivial, small, medium, large, and very large, respectively. Changes of DCPs and HRV for all participants at each group were used to calculate Pearson’s correlation in which values of 0.1–0.39, 0.4–0.69, and 0.7–0.9 were treated as weak, moderate and strong correlation, respectively. In this study, the alpha level for statistical significance was set at 0.05.

## Results

The pre-post changes of DCPs, FDV (i.e., nLF, nHF and LF/HF) and the MHR are presented in Table 2. In general, DCPs [F(2.71, 32.48) = 8.5, *p* < 0.01, η2 p = 0.42, large], nLF [F(3.55, 42.62) = 22, *p* < 0.01, η2 p = 0.65, large], the LF/HF [F(3.97, 47.7) = 30, *p* < 0.01, η2 p = 0.71, large] rather than the MHR [F(3.93, 47.14) = 1.75, *p* = 0.16, η2 p = 0.13, medium] considerably differed at frequencies of 5–35 Hz. DCPs were considerably lower at 5 Hz [−2.13 μV, t(84)= −3.82, *p* < 0.05, g = −1.03, large] than the ones of the CON group, but they substantially improved at 20 Hz [7.58 μV, t(84) = 4.31, *p* < 0.05, g = 1.16, large], 25 Hz [6.06 μV, t(84) = 2.66, *p* < 0.05, g = 0.71, large] and 35 Hz [5.66 μV, t(84) = 3.66, *p* < 0.05, g = 0.98, large]. Additionally, DCPs at 5 Hz [t(84) = −4.35, *p* < 0.01, g = −1.17, large], 20 Hz [t(84) = 5.27, *p* < 0.01, g = 1.42, very large], and 35 Hz [t(84) = 5.31, *p* < 0.01, g = 1.43, very large] were different from those in the SSE group.

**Table 2:** Pre-post changes of direct current potential, normalized low frequency power, normalized high frequency power, the ratio of low frequency power to high frequency power and the mean heart rate for acute whole-body vibration at frequencies of 5–35 Hz.

Group	DCP (μV)	nLF (%)	nHF (%)	LF/HF	MHR (beat)
SSE	1.26	0.09	−0.09	0.15	6.92
CON	1.64	0.03	−0.03	0.09	−7.61
5 Hz	−2.13^*,^^##^	−0.13^*,^^##^	0.13^*,^^##^	−0.37^##^	1.69
9 Hz	0.01	−0.01^#^	0.01^#^	−0.05	3.38
13 Hz	2.29	0.06	−0.05	0.16	5.15
20 Hz	7.58^*,^^##^	0.17^*,^^#^	−0.17^*,^^#^	0.51^#^	6.58
25 Hz	6.06^*,^^##^	0.25^**,^^##^	−0.25^**,^^##^	0.89^**,^^##^	8.46
35 Hz	5.66^*,^^##^	0.31^**,^^##^	−0.31^**,^^##^	1.23^**,^^##^	10.08

CON, control group; DCP, direct current potential; LF/HF, ratio of low frequency power to high frequency power; MHR, mean heart rate; nLF, normalized low frequency power; nHF, normalized high frequency power; SSE, static squat exercise; ^*^ *p* < 0.05, ^**^ *p* < 0.01, 5–35 Hz versus CON; ^#^ *p* < 0.05, ^##^ *p* < 0.01, 5–35 Hz versus SSE

As for the FDV, nLF at 5 Hz [−13%, t(84) = −2.31, *p* = 0.04, g = −0.62, medium], 20 Hz [17%, t(84) = 2.93, *p* < 0.05, g = 0.79, large], 25 Hz [25%, t(84) = 5.16, *p* < 0.01, g = 1.37, very large], and 35 Hz [31%, t(84) = 4.42, *p* < 0.01, g = 1.19, large] were substantially different from the value of the CON group. However, in comparison with participants of the SSE group, the follow-up post-hoc analysis indicated a considerable increase in nLF at 5 Hz [t(84) = −5.38, *p* < 0.01, g = −1.44, very large], and a significant decrease at 20 Hz [t(84) = 4.22, *p* = 0.04, g = 1.13, large], 25 Hz [t(84) = 6.27, *p* < 0.01, g = 1.61, very large], and 35 Hz [t(84) = 8.1, *p* < 0.01, g = 2.17, very large]. The opposite changes were found at different groups with respect to nHF. The LF/HF was significant at 5 Hz [−0.37, t(84) = −2.58, *p* < 0.05, g = −0.69, large] and 20–35 Hz [0.51, t(84) = 2.86, *p* < 0.05, g = 0.77, large] in contrast with the CON group. Furthermore, it was estimated that 30 Hz may be the frequency at which the LF/HF reached 1. On the other hand, a considerable change in the LF/HF at 5 Hz [t(84) = −4.79, *p* < 0.01, g = −1.29, very large], 20 Hz [t(84) = 3.71, *p* = 0.04, g =1, large], 25 Hz [t(84) = 6.94, *p* < 0.01, g = 1.86, very large], and 35 Hz [t(84) = 7.85, *p* < 0.01, g = 2.11, very large] were observed compared with the value of the SSE group. With respect to the MHR change, it was lower at the frequency of 5 Hz (1.69 beats/min) and 9 Hz (3.38 beats/min) in comparison with the CON group (−7.61 beats/min), the SSE group (6.92 beats/min) and frequencies at 35 Hz (10.08 beats/min). Nevertheless, no significant change of the MHR was found at either of the two groups (*p* > 0.05).

With regard to the correlation between nLF and DCPs, a moderate correlation at 13 Hz (r = −0.4) and a strong correlation at 5 Hz (r = 0.7) in contrast with weak correlation in other frequencies were found (Table 3).

**Table 3:** Correlations between normalized low frequency power, normalized high frequency power and direct current potentials for acute whole-body vibration at frequencies of 5–35 Hz.

Group	Pearson correlation coefficient		*p*	Effect
	nLF and DCP	nHF and DCP		
5 Hz	0.7	−0.7	0.008^**^	strong
9 Hz	0.1	0.1	0.989	weak
13 Hz	−0.4	0.4	0.213	moderate
20 Hz	−0.1	0.1	0.830	weak
25 Hz	−0.2	0.2	0.579	weak
35 Hz	0.2	−0.2	0.424	weak

nLF, normalized low frequency power; nHF, normalized low frequency power; DCP, direct current potential; ^*^ *p* < 0.05, ^**^ *p* < 0.01

## Discussion

The effect of WBV at various frequencies on HRV and BE may indicate a potential role in previously reported benefits, but this problem has not been clearly elucidated. The main findings of the present study are that WBV at 20–35 Hz can activate the SNS and improve BE, whereas WBV frequency of 5 Hz mainly facilitates the PNS activation and decreases BE. HRV and BE have a synchronous reaction at 5 Hz of acute WBV intervention.

As for the response of the SNS to acute WBV, nLF and the LF/HF continuously improved with the increase of the frequency from 5 Hz to 35 Hz, suggesting that the activation of the SNS was sensitive to the improvement of WBV frequency. This finding was consistent with a previous study observing that 31.5 Hz rather than 16 Hz of WBV caused the most considerable effects on the SNS when participants were sitting on the WBV device (Ando and Noguchi, 2003). Additionally, significant activations of the SNS observed at frequency of 20–35 Hz with large to very large effect size possibly meant that WBV at 20 Hz may be the minimal intensity to affect the SNS. On the other hand, it was around 30 Hz at which the LF/HF exceeded 1, partly indicating that 30 Hz was probably the minimum frequency to keep the SNS dominant in the WBV intervention. In line with our hypothesis, another major finding was that acute WBV at 5 Hz was considerably effective in activating the PNS. Medium (g = 0.62) and very large (g = 1.44) effect size of nLF were found at 5 Hz when compared with participants in the CON and SSE groups, respectively, suggesting that 5-Hz WBV intervention performed in a standing position was capable of modulating the ANS to the PNS dominance in contrast with blank intervention or static squat exercise. Previous studies found that WBV at 5 Hz, 15 Hz (Liu et al., 2021), but not 20 Hz (Sañudo et al., 2013) could promote the activation of the PNS after intense exercises when volunteers adopted a seating posture. In addition, WBV at 1.8 Hz was more effective than 6 Hz in activating the PNS in a driving context (Jiao et al., 2004). This evidence suggests that the frequency of 20–35 Hz mainly influenced the SNS, but the PNS was affected by relatively low frequency (probably around 5 Hz). As the activation of the SNS and the PNS was responsible for increasing and decreasing the heart rate, respectively, changes in nLF and the LF/HF could be used to explain the improvement and the decline in the MHR at 20−25 Hz and 5 Hz. In practice, WBV frequency around 5 Hz and 20 Hz could be considered to guide the intervention targeted at HRV and BE.

For BE changes, the main finding was that the frequency from 20 Hz to 35 Hz increased BE, while 5 Hz WBV reduced BE. With the enhancement of frequency at 5–20 Hz, a continuous increase in BE was found, suggesting that there were incremental responses at the level of the central nervous system facing the improved vibration load. This increase may provide evidence for the alteration of cognitive tasks in a previous study (Regterschot et al., 2014). On the other hand, DCPs reached a significant level at 20 Hz in comparison to participants in the CON group, partly indicating that 20 Hz may be the minimal frequency to induce a significant effect on BE, which was partly in line with another study that the motor network and prefrontal cortical areas of healthy adult males could be activated by WBV at 27 Hz rather than 10 Hz (Choi et al., 2019). Hence, frequency of at least 20 Hz may be necessary if the intervention purpose is to increase BE. Nevertheless, there was an unexpected finding that DCPs had a non-significant decline at 25 Hz and 35 Hz following the peak level of 20 Hz. Indeed, it was previously found that 30 Hz appeared to have a greater effect on improving performance than 35 Hz, 40 Hz and 50 Hz (Bedient et al., 2009). Hence, frequency around 20 Hz may be a turning point after which the phenomenon of central fatigue or overload may gradually appear and accordingly weaken the output of neuromuscular capacity. There was a pronounced reduction in DCPs at 5 Hz in comparison to the CON and SSE groups, suggesting that WBV frequency at 5 Hz could reduce BE. This finding may be a novelty as fewer evidence has been reported in the area of sport science and medicine regarding effects of WBV at around 5 Hz on BE. In the driving context, frequency at 4–7 Hz has shown the capability of causing sleep-inducing effects such as improved drowsiness or sleepiness, reduced alertness or attention, and this effect was frequency-dependent (Bhuiyan et al., 2022; Zhang et al., 2018). Although the position that participants adopted in the present study was different from the posture in a driving setting, reduced BE in those studies indicated that WBV frequency around 5 Hz may be sufficient to reduce BE. In other words, WBV around 5 Hz tended to “loosen up” instead of “cheering up” the human body.

Regarding the correlation between HRV and BE, the correlation between nLF and DCPs (r = 0.7) at 5 Hz was strong in contrast with other frequencies, indicating that WBV at frequency of 5 Hz had a strong synchronous effect on reducing BE and the SNS. In fact, the observed correlation between HRV and BE was partly in agreement with a previous study revealing that PNS activation was considerably correlated with the activity of the anterior cingulate cortex in the short-term meditation intervention (Tang et al., 2009). Therefore, it could be speculated that the correlation between HRV and BE might be modulated by external mechanical vibration stimulation and this effect was dependent on the frequency of WBV, in which lower frequency (around 5 Hz) rather than higher frequency was more effective.

Despite the effects of WBV on HRV and BE, the underlying mechanism has not been clearly elucidated, especially the role of different frequencies. It could be hypothesized that both neuronal and humoral regulation may be involved. The phenomenon of neuronal entrainment enables the interaction between rhythms of BE and rhythms of an external environment (e.g., motor production and sensory perception) in complex ways (Lakatos et al., 2019). An animal study found that there was rhythmically firing in the primary somatosensory cortex of monkeys when exposed to palmar vibration and these activations might evoke intermittent oscillations in other cortical neurons to regulate cortical population oscillations (Lebedev and Nelson, 1995). In this sense, it may be plausible that rhythm of brain activity can be modulated by external sinusoidal vibration stimulation. WBV at 5–35 Hz may be regarded as one of external rhythmic somatosensory input to entrain the rhythms of BE and accordingly modulate BE and sympathetic outflow mediated by the central nervous system. Additionally, the effect of neuronal entrainment may be especially strong in external frequency below 10 Hz (Lakatos et al., 2019), which may be used to partly explain why there were significant changes at 5 Hz instead of higher frequencies concerning HRV, BE and correlation between HRV and BE. WBV intervention at 5 Hz may be sufficient to entrain cortical theta bands and prepare the brain to enter the state of sleep from wakefulness (Bhuiyan et al., 2022). On the other hand, humoral regulation may also play an important role in effects of WBV on HRV and BE. Nitric oxide could modulate baroreflex-mediated cardiac vagal control demonstrated by HRV and baroreflex sensitivity in humans (Chowdhary et al., 2000). Besides, it may also be viewed as essential signaling molecule for integrating sensory and homeostatic-related cues to endow brain neuronal networks with the ability of making adjustments to key bodily functions (Chachlaki and Prevot, 2020). During WBV intervention, blood flow velocity and muscle perfusion were improved to consequently induce the release of nitric oxide probably through the increased shear stress on the endothelial cell (Aoyama et al., 2019). In addition, this effect was possibly influenced by the frequency. In this case, the release of nitric oxide caused by WBV may be regarded as a role of humoral regulation to change HRV and BE. Nonetheless, it is still warranted to investigate the specific pathway with which this effect works.

There are a number of limitations in this study. First, as participants were young physically active students, it may be difficult to generalize these results to other populations. Second, this study did not explore frequencies lower than 5 Hz and higher than 35 Hz because of the unavailability of the equipment, which possibly limited the understanding of the effects of WBV at these frequencies. To comprehensively evaluate responses of the human body to WBV at different frequencies, it is recommended to extend the spectrum of vibration frequency in future studies. Third, DCPs can reflect the excitability of the cerebral cortex and subcortical structures, but it may be limited in detecting the specific neural oscillatory patterns. In this regard, other brain measures (e.g., electroencephalography) are still suggested to explore the association between external mechanical vibration stimulation and neural responses.

## Conclusions

Acute WBV at frequencies of 5–35 Hz had different effects on heart rate variability and brain excitability. The frequency of 20–35 Hz effectively enhanced the activation of the sympathetic nervous system and improved brain excitability, while WBV at 5 Hz principally affected the parasympathetic nervous system and reduced brain excitability. Heart rate variability and brain excitability had synchronous reaction at 5 Hz of WBV. In practice, the frequency of WBV at 5–35 Hz could be manipulated depending on the intervention target at heart rate variability and brain excitability.
